# Comparative analysis of codon usage patterns in the chloroplast genomes of nine forage legumes

**DOI:** 10.1007/s12298-024-01421-0

**Published:** 2024-03-09

**Authors:** Mingkun Xiao, Xiang Hu, Yaqi Li, Qian Liu, Shaobin Shen, Tailing Jiang, Linhui Zhang, Yingchun Zhou, Yuexian Li, Xin Luo, Lina Bai, Wei Yan

**Affiliations:** 1 Tropical and Subtropical Cash Crops Research Institute, Yunnan Academy of Agricultural Sciences, Baoshan, Yunnan China; 2 Tropical Eco-agricultural Research Institute, Yunnan Academy of Agricultural Sciences, Yuanmou, Yunnan China

**Keywords:** Forage legume, Codon usage bias, Chloroplast genome, Phylogenetic relationships

## Abstract

*Leguminosae* is one of the three largest families of angiosperms after *Compositae* and *Orchidaceae*. It is widely distributed and grows in a variety of environments, including plains, mountains, deserts, forests, grasslands, and even waters where almost all legumes can be found. It is one of the most important sources of starch, protein and oil in the food of mankind and also an important source of high-quality forage material for animals, which has important economic significance. In our study, the codon usage patterns and variation sources of the chloroplast genome of nine important forage legumes were systematically analyzed. Meanwhile, we also constructed a phylogenetic tree based on the whole chloroplast genomes and protein coding sequences of these nine forage legumes. Our results showed that the chloroplast genomes of nine forage legumes end with A/T bases, and seven identical high-frequency (HF) codons were detected among the nine forage legumes. ENC-GC3s mapping, PR2 analysis, and neutral analysis showed that the codon bias of nine forage legumes was influenced by many factors, among which natural selection was the main influencing factor. The codon usage frequency showed that the *Nicotiana tabacum* and *Saccharomyces cerevisiae* can be considered as receptors for the exogenous expression of chloroplast genes of these nine forage legumes. The phylogenetic relationships of the chloroplast genomes and protein coding genes were highly similar, and the nine forage legumes were divided into three major clades. Among the clades *Melilotus officinalis* was more closely related to *Medicago sativa*, and *Galega officinalis* was more closely related to *Galega orientalis*. This study provides a scientific basis for the molecular markers research, species identification and phylogenetic studies of forage legumes.

**Supplementary Information:**

The online version contains supplementary material available at 10.1007/s12298-024-01421-0.

## Introduction

Forage legume is an important part of global grassland agriculture. Compared with grass forage, forage legume has some unique advantages, such as exhibiting symbiosis with *rhizobium*, which is beneficial to reduce nitrogen fertilizer input in low-nitrogen land (Phelan et al. 2015). With rising energy and fertilizer prices, stringent environmental regulations, and high feed costs, the importance of forage legume in ruminant production agricultural systems will be enhanced (Lüscher et al. 2014; Van Grinsven et al. 2013) At the same time, adding forage legume to ruminant daily feed can improve the digestibility and metabolic energy of feed to meet the protein requirements of animals (Rochon et al. 2004). Currently, the number of legumes used as forage worldwide is currently unknown; the Food and Agriculture Organization of the United Nations, online animal feed information base (http://www.feedipedia.org/content/feeds?category=13594) lists only 153 forage legumes. However, only a very small fraction of forage legume is widely considered to be of global commercial importance (Gustavo and Timothy 2017; Phelan et al. 2015; Peyraud et al. 2009). In this study, we compared the codon preferences of the chloroplast genomes of seven perennial and two annual forage legumes in world grassland agriculture.

Chloroplasts are energy converters unique to higher plants and some algae (Zhang et al. 2012). Plants use solar energy, water, minerals, and carbon dioxide through photosynthesis to synthesize organic compounds to sustain life (Dobrogojski et al. 2020). In addition, chloroplasts are essential carriers for the synthesis of amino acids, nucleotides, plant hormones, fatty acids, and vitamins. These metabolites play an important role in plant stress response and information transmission (Daniell et al. 2016). Compared with those of nuclear and mitochondrial genomes, the structure and function of chloroplast genome are characterized by small size, high conservation, simple structure, and single-parent inheritance, which have great advantages in genetic transformation (Wang et al. 2023), thus chloroplast attracting the attention of many scholars in recent years.

Codons are the link between amino acids, proteins, and genetic material in living organisms and play an irreplaceable role in transmitting genetic information. Living organisms have 64 codons, 3 of which are termination codons, and the other 61 are translated into 20 amino acids. Except methionine and tryptophan that have unique codons, the rest of the amino acids usually have 2–6 corresponding codons, and codons coding for the same amino acid are called synonymous codons. Genes from different species or within the same species exhibit different codon usage preference patterns, and the phenomenon of uneven usage of synonymous codons that is prevalent in organisms, is known as codon bias (Wang et al. 2020; Plotkin and Kudla 2011). Codon bias to some extent can reflect the origin of species or genes, the evolutionary process, and the mode of mutation. In animals, it occurs mainly from mutational pressure in microorganisms and from natural selection; but for plants, it is influenced by natural selection and mutational pressure (Bulmer 1991; Baeza et al. 2015; Camiolo et al. 2015; Boel et al. 2016). High-throughput sequencing technologies have allowed the enrichment of chloroplast genome databases and the analysis of genome codon preference in a variety of organisms. Example include the comparative analysis of codon bias in the chloroplast genomes of *Theaceae* species (Wang et al. 2022), comparative analysis of codon usage patterns in the chloroplast genomes of ten *Epimedium* species (Wang et al. 2023), analysis of codon usage bias of chloroplast genomes in *Gynostemma* species (Zhang et al. 2021), and analysis of the codon usage bias of chloroplast genes in *Oryza* species (Chakraborty et al. 2020). Many reports are available on the chloroplast genomes of *Leguminosae*, but only a few have focused on their codon bias. Studying the codon usage patterns in plants is necessary to construct stable transgenic systems.

In this study, the codon usage preference characteristics of chloroplast genes of nine forage legumes were analyzed from the perspective of coding sequences and explored the reasons for their formation. We have also analyzed several important parameters of codon usage patterns, including GC content at three positions (GC1, GC2, and GC3), effective number of codons (ENC), relative synonymous codon usage (RSCU), and relative frequency of synonymous codon usage (RFSC). In addition, the codon usage frequencies of these nine forage legumes were compared with four model organisms i.e.,  *Arabidopsis thaliana*, *Nicotiana tabacum*, *Escherichia coli*, and *Saccharomyces cerevisiae*. This work provides a basis for further improving the exogenous gene expression efficiency of the nine forage legumes. The findings reveal the role of usage patterns and sources of variation in influencing the codon usage bias of the chloroplast genomes of different forage legumes during the evolutionary process, and provide a reference for the verification of gene function, genetic evolution and, transgenic engineering of chloroplast genomes.

## Materials and methods

### Genomes and coding sequences data acquisition and filtering of nine forage legumes

The chloroplast genomes and coding sequences (CDS) of nine forage legumes were downloaded from the National Center for Biotechnology Information database (NCBI, https://www.ncbi.nlm.nih.gov/, accessed on 15 May 2023). For ensure the accuracy of the experimental results, the CDS of the nine plants should meet the following conditions: (1) the number of bases in the CDS of each plant should be an integer multiple of 3; (2) the length of each CDS must be ≥ 300 bp (Wang et al. 2020); (3) the CDS should be of high quality with recognized bases, in other words, they contain only the bases of A, T, G, and C; (4) the sequence starts with the start codon ATG and ends with the stop codon TAA, TAG or TGA; 5) the CDC series does not contain intermediate termination codons (He et al. 2016; Li et al. 2016;). The GC content of three positions (GC1, GC2, and GC3) was calculated using the CUSP program in EMBOSS explorer (https://www.bioinformatics.nl/emboss-explorer/). Detailed information on the complete chloroplast genome and gene annotation of *Trifolium repens*, *Melilotus officinalis*, *Galega orientalis*, *Clitoria ternatea*, *Astragalus laxmannii*, *Galega officinalis*, *Pisum sativum*, *Stylosanthes guianensis* and *Medicago sativa* were shown in Table 1.

**Table 1:** Genetic characterization of chloroplast genomes of nine forage legumes

Species	Accession No	CDSs number (before processing)	CDSs number (after processing)	L-aa	GC1%	GC2%	GC3%	Average GC at three locations%
*Trifolium repens*	NC_024036.1	75	50	20,048	45.67	37.47	27.99	37.04
*Melilotus officinalis*	NC_070051.1	76	44	15,855	46.25	37.85	27.00	37.04
*Galega orientalis*	NC_069214.1	73	47	16,817	46.26	37.87	26.54	36.89
*Clitoria ternatea*	NC_047365.1	81	50	20,331	44.11	36.55	25.98	35.55
*Astragalus laxmannii*	NC_052923.1	75	48	18,218	45.47	37.51	26.50	36.49
*Galega officinalis*	NC_051885.1	77	48	19,808	45.23	37.03	27.21	36.49
*Pisum sativum*	NC_014057.1	74	52	20,439	45.57	37.01	28.00	36.86
*Stylosanthes guianensis*	NC_058691.1	82	52	20,891	45.34	37.76	29.17	37.42
*Medicago sativa*	NC_042841.1	76	51	20,649	45.44	37.14	26.93	36.50

L-aa means the total number of amino acids; GC1, GC2, and GC3 indicate the GC content at the first, second, and third codon positions

### Analysis of RSCU, RFSC, and HF

RSCU is a statistical index that measures the ratio of the relative usage frequency of each synonymous codon (Wang et al. 2022). RSCU greater than 1, indicates that the codon is used more frequently than other synonymous codon frequencies and is positively biased for a specific amino acid. RSCU less than 1, indicates that the codon is used less frequently than other synonymous codon frequencies and is negatively biased for a specific amino acid. RSCU of 1, indicates that the codon is not biased for a specific amino acid and is selected equally or randomly in the RNA transcript. Synonymous codons with RSCU values greater than 1.6 and less than 0.6 are considered overrepresented and underrepresented, respectively (Butt et al. 2014). RSCU is calculated using the formula of Sharp and Li (1986) as follows:

$${\text{RSCU = }}\frac{{{\text{X}}_{{{\text{ij}}}} }}{{\mathop \sum \nolimits_{{\text{j}}}^{{{\text{n}}_{{\text{i}}} }} {\text{X}}_{{{\text{ij}}}} }}{\text{n}}_{{\text{i}}}$$

where X_*ij*_ denotes the number of *j*th codons encoding the *i*-th amino acid, and *n*_*i*_ denotes the number of synonymous codons encoding the *i*-th amino acid, with the value ranging from 1 to 6.

RFSC is the ratio of the number of one codon to the number of synonymous codons in the actual observation, reflecting the usage frequency of each synonymous codon. It is calculated using the formula of Sharp and Li (1986) as follows:

$${\text{RFSC = }}\frac{{{\text{X}}_{{{\text{ij}}}} }}{{\mathop \sum \nolimits_{{\text{j}}}^{{{\text{n}}_{{\text{i}}} }} {\text{X}}_{{{\text{ij}}}} }}$$

where X_*ij*_ denotes the number of *j-*th codons encoding the *i*-th amino acid. RFSC more than 60% or more than 0.5 times the average frequency of the synonymous codon indicates that the synonymous codon is a HF codon (Zhou 2007).

### Analysis of the optimal codons

The high and low expression datasets of genes were established according to the ENC value of each gene. RSCU and ΔRSCU were then calculated using Codon W 1.4.2 software, and codons that satisfy the conditions of RSCU > 1 and ΔRSCU > 0.08 were defined as optimal codons (Romero et al. 2000).

### Comparative analysis of codon usage frequency

For in-depth analysis of the codon usage patterns of nine forage legumes, the codon usage frequency data for the genomes of four model organisms, *Arabidopsis thaliana* (http://www.kazusa.or.jp/codon/cgi-bin/showcodon.cgi?species=3702), *Nicotiana tabacum* (http://www.kazusa.or.jp/codon/cgi-bin/showcodon.cgi?species=4097), *Escherichia coli* (http://www.kazusa.or.jp/codon/cgi-bin/showcodon.cgi?species=199310), and *Saccharomyces cerevisiae* (http://www.kazusa.or.jp/codon/cgi-bin/showcodon.cgi?species=4932), were downloaded from the Codon Usage Database (http://www.kazusa.or.jp/codon). The codon usage frequencies of the genomes of the nine forage legumes were compared with those of the four model organisms to investigate differences in codon usage patterns. In addition, the ratio of codon usage frequency of nine forages legumes to four model organisms was calculated. A ratio of ≥ 2 or ≤ 0.5, indicates that the two organisms have a large difference in codon usage bias and should not be selected as gene heterologous expression receptors (Pan et al 2013). A ratio between 0.5 and 2, indicates that they have a high degree of similarity in codon usage patterns and could be considered as gene heterologous expression receptors (Zhou et al. 2007a, b).

### Analysis of ENC-plot

ENc is often used to analyze the degree of synonymous codon usage preference in the genes or genomes of specific species, its value ranges 20–61, in which a small value indicates a strong codon usage preference (Wu et al. 2018). GC3s refers to the ratio of the G + C content of the third position of the codon of the CDS sequence to the total number of bases of genes other than Met and Trp. The ENC-plot was plotted with GC3s as the horizontal coordinate and ENC as the vertical coordinate to reveal the factors influencing the codon usage pattern of a gene or genome. When mutational pressure plays a major role in codon usage patterns, the ENC values lie on or near the expected curve. When natural selection and other factors play a major role in codon usage patterns, the ENC values lie well below the expected curve (Wright 1990).

$${\text{ENC = 2 + S + }}\frac{{{29}}}{{{\text{S}}^{{2}} { + }\left( {\text{1 - S}} \right)^{{2}} }}$$

### Parity rule 2 bias plot (PR2-plot) analysis

PR2-plot was used to analyze the usage and relationship of the four bases at position 3 of the codon encoding amino acids. Scatter plots were drawn with A3/(A3 + T3) as the vertical coordinate and G3/(G3 + C3) as the horizontal coordinate. The center of the plot refers to the position where A=T or G=C. In general, the ratio of A/T and C/G in codons of a gene or genome is balanced under a single mutational pressure (Xiang et al. 2015). The ratios vary considerably when the codon usage preferences have influenced by natural selection and other factors (Sueoka 1999a, b; Sueoka 1995).

### Analysis of neutrality plot

Neutrality analysis was used to study the effects of mutational pressure and natural selection on codon preference (He et al. 2016). Here, y-axis GC12 represents the average of the GC content of the first and second codon positions, and x-axis GC3 represents the GC content of the third codon position. If GC12 and GC3 are unaffected by mutation pressure, then the points should be distributed along a diagonal (slope of 1). Conversely, if GC12 is completely nonneutral to GC3, then the points should be on the parallel lines of the horizontal coordinates (slope of 0). There trends suggest that codon preferences are affected by natural selection. When the regression coefficients converge or are equal to 1 and the correlation is significant, then mutational pressure plays a major role in the gene (Sueoka 1999a, b).

### Correspondence analysis (COA)

COA is a statistical method used to analyze the relationship between variables and samples (Romero et al. 2003). Each CDS was represented as a 59-dimensional vector (excluding codons encoding the Met and Trp proteins and the three-stop codons) (Sharp and Li 1986), and each dimension corresponded to the RSCU of each synonymous codon (Wei et al. 2014). R programming language was used to draw the relationship chart of axis 1, axis 2, and the codon utilization index (the GC content of GC3s, the codon adaptation index (CAI), ENC, and L_aa) to further investigate the components affecting codon utilization preference.

### Phylogenetic tree construction of nine forage legumes

To clarify the interspecific affinities and phylogenetic position of nine forage legumes, the nine forage legumes were used as ingroup, while *Rosa cymose*, *Broussonetia kaempferi*, *Fragaria viridis*, and *Elatostema dissectum* were selected as outgroup to jointly construct a phylogenetic tree. These chloroplast genomes and CDSs were subjected to multiple sequence comparisons and trimmed by MEGA 7.0 software (Shahzadi et al. 2020; Chi et al. 2023). Phylogenetic trees were constructed by neighbor-joining (NJ), and set 1000 times of Bootstrap test (BS) for the confidence of each clades, and all other parameters were set as default values. Evolutionary trees were beautified with Figtree software (http://tree.bio.ed.ac.uk/software/figtree/).

## Results

### Characteristics of codon base composition

The CDSs of the nine forage legumes processed by the Perl script contained 50, 44, 47, 50, 48, 48, 52, 52, and 51 (Table 1). Codon usage patterns are closely related to GC content (Shackelton et al. 2006), so we calculated the GC content of the three loci of codons GC1, GC2, and GC3 for each of the nine forage legumes species. We found that the contents of GC1, GC2, and GC3 and the average content of GC at the three loci of the nine forage legumes grasses were less than 40.00%. The order in each species was GC1 > GC2 > GC3, indicating that all nine forage legumes chloroplast genomes are inclined to use codons ending in A/T bases. In addition, the average GC content was the same for the three positions of *T. repens* and *M. officinalis* (37.04), and the average GC content was the same for the three positions of *A. laxmannii* and *G. officinalis* (36.49), but the contents of the other five species of forage legumes varied slightly (35.55–37.42; Table 1). The chloroplast genome codon preference for the use of A/T endings has been reported in 10 species of *Epimedium* species (Wang et al. 2023), 40 *Theaceae* species (Wang et al. 2022), and 18 *Oryza* species (Chakraborty et al. 2020).

### RSCU and RFSC

To further understand the synonymous codon usage pattern and the preferences for G/C terminal codons and A/T terminal codons, we performed an RSCU analysis of codons in nine forage legumes. The results showed that the number of optimal codons (RSCU > 1) in the chloroplast genomes of the nine forage legumes was 30, except for *C. ternatea* that had 31. Among these, 29 of their codons ended with A/T (13 A and 16 T) except *C. ternatea* with 30 (14 A and 16 T). These values accounted for 96.68% of the total number of preferred codons, which was more than the number of codons ended with G/C. In summary, the nine forage legumes chloroplast genes showed a relatively great preference for A/T ended codons.

**Fig. 1:**
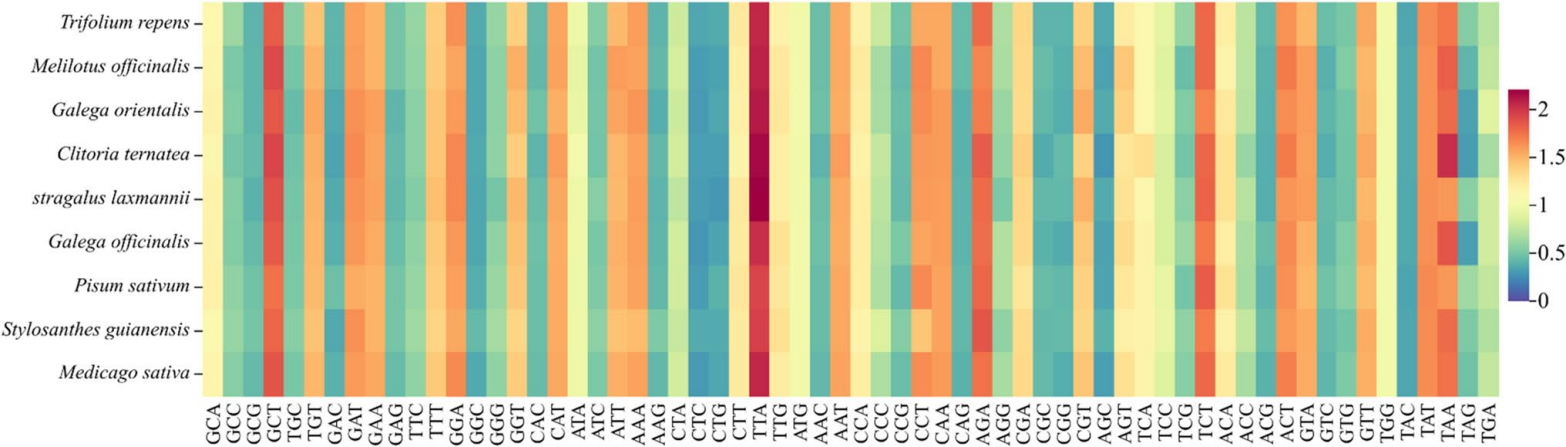
Relative synonymous codon usage of chloroplast genes in nine forage legumes (blue to red indicates codons with low to high RSCU values)

The range of variation in the RSCU values of the nine forage chloroplast genomes was close (Table S1). The TTA of codon encoding Leu had the highest RSCU (2.21), and the AGC of codon encoding Ser had the lowest RSCU (0.27). The following 15 commonly used HF codons were found in the chloroplast genomes of the nine forage legumes: GCT, GAT, GAA, GGA, CAT, TTA, ATG, AAT, CAA, AGA, TCT, GTT, TGG, TAT, and TAA (Fig. 1). In addition, codon overexpression (RSCU > 1.6) was found in all nine forage legumes, such as 9 in *P. sativum*, *M. officinalis*, and *S. guianensis*; 10 in *G. officinalis*; 11 in *C. ternatea* and *A. laxmannii*; 12 in *M. sativa*; and 13 in *C. ternatea* and *G. orientalis*. It is noteworthy that seven codons (GCT, TTA, AGA, TCT, ACT, TAT, and TAA) were overexpressed in all nine forages.

### Determination of optimal codons

The high and low expression datasets of the chloroplast genes of nine forage legumes were established according to the ENC value of each CDS, and the optimal codons were screened by RSCU > 1 and ΔRSCU > 0.08. The results are shown in Table 2, with 149 optimal codons found for the nine forage legumes. Among them, 50 codons ended in A, 95 ended in T, and 4 ended in G. GCT, ACT, CCT, GGT, and TCT were the common optimal codons for the nine forage legumes.

**Table 2:** Optimal codons of chloroplast genomes of nine forage legumes

Species	Number	Optimal codon
*Trifolium repens*	20	TAA, GCT, TCT, CGT, CCT, AGA, TGT, ACT, GTT, GTA, TAT, CTT, CAA, GGT, GAA, TTT, GAT, ATT, TTA, GGA
*Melilotus officinalis*	14	CCT, CGT, GCT, GGT, ACT, TTA, TCT, TAA, ATT, TGT, CAA, GTA, GAT, TAT
*Galega orientalis*	14	GCT, ACT, CGT, TTG, CCT, GTT, GGT, CTT, GAT, TTA, ATT, AGT, CAA, GTA
*Clitoria ternatea*	14	ACT, GCT, TTA, TAA, GGT, TCT, CGA, TGT, CCT, GAA, GTT, AGA, TTT, TTG
*Astragalus laxmannii*	15	GCT, GTT, CGA, CTT, TAA, ACT, TCA, CAA, TTA, AGA, CCT, GGT, TCT, CCA, CAT
*Galega officinalis*	14	GCT, CGT, GTT, ACT, TTG, CCT, AGT, GGT, TTA, ATT, TTT, TGT, TCT, TAT
*Pisum sativum*	26	TTA, GGA, TAA, TCT, GAT, GCT, GTT, CAA, CCT, CGT, ACA, ATT, AGA, CTT, ACT, TGT, TAT, GAA, TTT, CCA, AAA, TTG, GTA, AGT, AAT, GGT
*Stylosanthes guianensis*	17	TTA, GCT, CGT, GAA, ATT, CAA, GGT, CGA, TAT, ACT, TCT、CCT,, TGT, TTT、AAA,, AGA, CCA
*Medicago sativa*	15	TAA, GCT, CTT, CGT, GTT, CCT, TCT, ACA, AGA, GGT, CAA, GAA, GCA, TAT, ACT

### Codon usage frequency

Considering the differences in the codon usage preference of chloroplast genes of the nine forage legumes, we have compared the genomic codon usage frequencies of the nine forage grasses with those of the four model species in this study (Table 3). The results showed differences in the number of codons between the nine forage legumes and the four model species, in the descending order, *E. coli* (25–29), *A. thaliana* (13–19), *N. tabacum* (9–14), and *S. cerevisiae* (9–11). It is noteworthy that *G. orientalis*, and *A. laxmannii* had the same number of differential codons as the four model species. *G. officinalis*, *S. guianensis*, and *M. sativa* had the same number of differential codons with *A. thaliana*, *N. tabacum*, and *S. cerevisiae*. The *M. officinalis* and *G. officinalis* had the same number of differential codons with *E. coli* and *S. cerevisiae*. Only in terms of codon usage frequency, the relatively high codon usage differences between the nine forage legumes and *E. coli* can theoretically be excluded as heterologous expression receptors, the *N. tabacum* and *S. cerevisiae* can be considered receptors for the exogenous expression of chloroplast genes in the nine forage legumes.

**Table 3:** Comparison of codon usage frequency between nine forage legumes and four model species

Species	Segment	*Arabidopsis thaliana*	*Nicotiana tabacum*	*Escherichia coli*	*Saccharomyces cerevisiae*
*Trifolium repens*	≥ 2 or ≤ 0.5	16	11	25	9
	0.5–2	48	53	39	55
*Melilotus officinalis*	≥ 2 or ≤ 0.5	12	9	26	10
	0.5–2	44	55	38	54
*Galega orientalis*	≥ 2 or ≤ 0.5	16	14	27	11
	0.5–2	48	50	37	53
*Clitoria ternatea*	≥ 2 or ≤ 0.5	19	14	29	12
	0.5–2	45	50	35	52
*Astragalus laxmannii*	≥ 2 or ≤ 0.5	16	14	27	11
	0.5–2	48	50	37	53
*Galega officinalis*	≥ 2 or ≤ 0.5	16	12	26	10
	0.5–2	48	52	38	54
*Pisum sativum*	≥ 2 or ≤ 0.5	13	10	26	8
	0.5–2	51	54	38	56
*Stylosanthes guianensis*	≥ 2 or ≤ 0.5	16	12	25	10
	0.5–2	48	52	38	54
*Medicago sativa*	≥ 2 or ≤ 0.5	16	12	27	10
	0.5–2	48	52	37	54

### Source analysis of variation in codon usage

#### ENC-plot

**Fig. 2:**
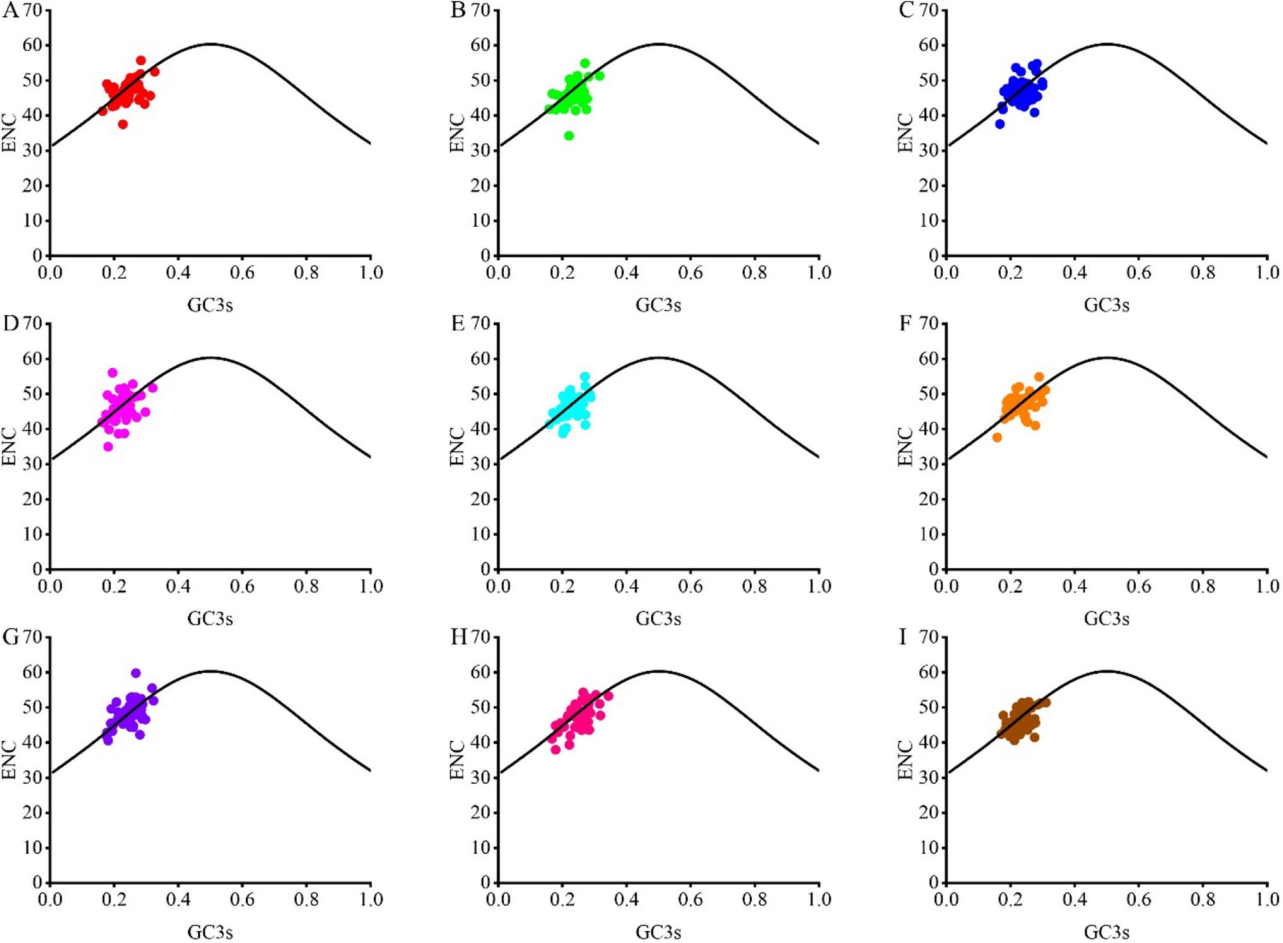
ENC-plot analysis in the chloroplast genomes of nine forage legumes (**A** *T. repens*, **B** *M. officinalis*, **C** *G. orientalis*, **D** *C. ternatea*, **E** *A. laxmannii*, **F** *G. officinalis*, **G** *P. sativum*, **H** *S. guianensis*, **I** *M. sativa*)

The effect of GC3s on codon preference was explored by drawing an ENC-GC3s plot to analyze the codon usage variation of chloroplast genes in the nine forage legumes (Fig. 2). As shown in the figure, the distribution of ENC-GC3s plots was relatively similar in the nine forage legumes. A small number of genes were located above or near the expected curve, suggesting that the mutational pressure had some effect on codon usage patterns. Most of the genes were located in the lower part of the expected curve, suggesting that natural selection plays an important role in the formation of codon usage bias.

#### PR2-plot

**Fig. 3:**
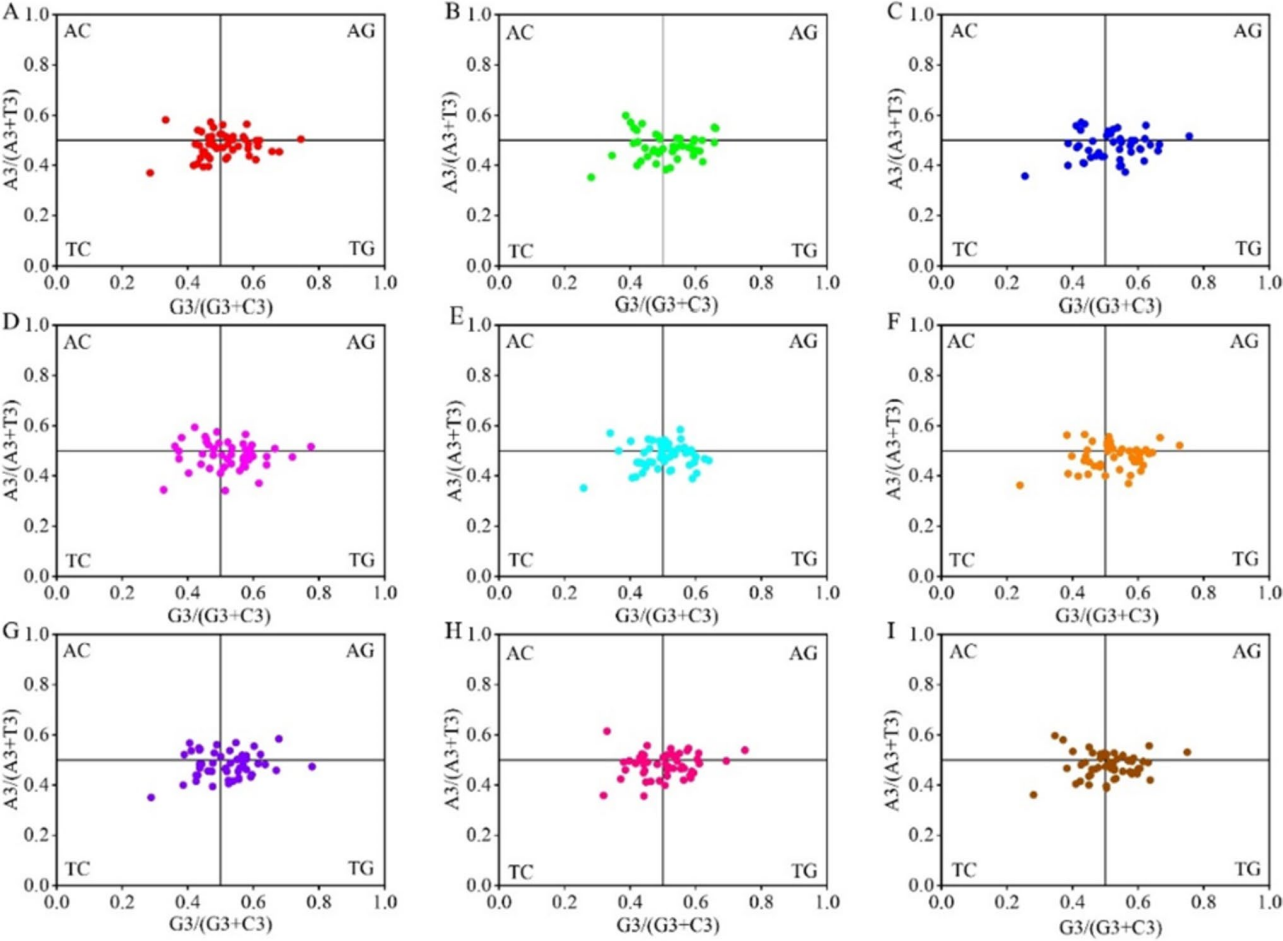
PR2-polt analysis of the chloroplast genomes of nine forage legumes (**A** *T. repens*, **B** *M. officinalis*, **C** *G. orientalis*, **D** *C. ternatea*, **E** *A. laxmannii*, **F** *G. officinalis*, **G** *P. sativum*, **H** *S. guianensis*, **I** *M. sativa*)

The usage between A/T and G/C at codon position 3 was analyzed to understand the effects of mutational and selection pressures on codon usage in the chloroplast genomes of the nine forage legumes (Fig. 3). If the frequencies of nucleotides A and T are equal to the frequencies of C and G in codon position 3, then the codon usage bias is only affected by mutational pressure (Kawabe and Miyashita 2003). On the contrary, the effect of natural selection would show inequality in the use of A and T bases and G and C bases. Observation of Fig. 3 shows that most genes in *T. repens* and *A. laxmannii* were distributed in the lower left region, indicating that T is used more frequently than A at codon position 3, and C is used more frequently than G. Most of the genes in the remaining seven forage legumes were distributed in the lower right region, indicating that T is used more frequently than A at codon position 3, and G is used more frequently than C. Therefore, the frequency of A/T and G/C use in the chloroplast genomes of the nine forage legumes were unbalanced, and natural selection plays an important role in the codon usage bias in the chloroplast genes of the nine forage legumes.

#### Neutrality plot

**Fig. 4:**
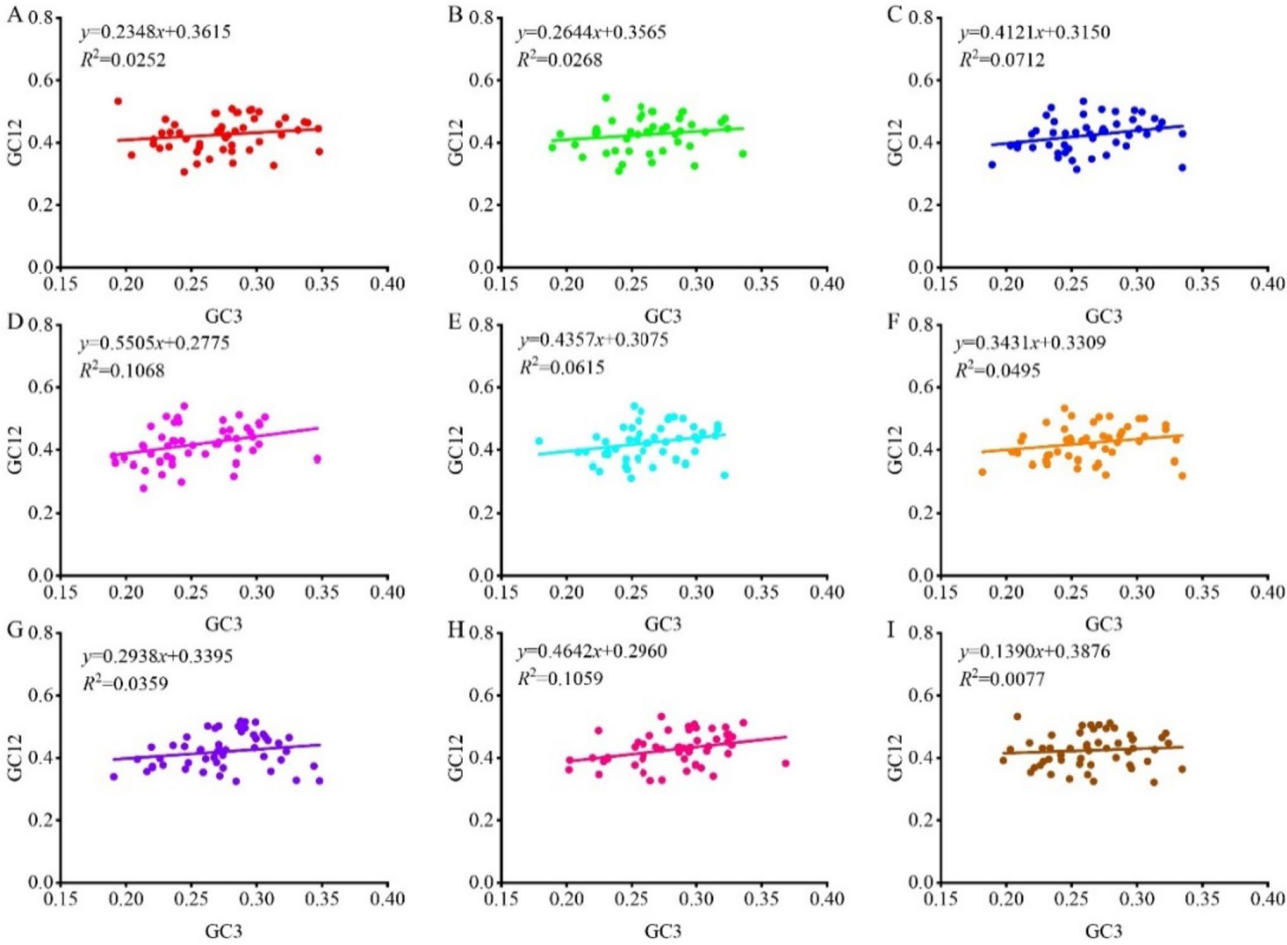
Neutrality plot analysis in the chloroplast genomes of nine forage legumes (**A** *T. repens*, **B** *M. officinalis*, **C** *G. orientalis*, **D** *C. ternatea*, **E** *A. laxmannii*, **F** *G. officinalis*, **G** *P. sativum*, **H** *S. guianensis*, **I** *M. sativa*)

The chloroplast genes were subjected to neutral analysis (regression analysis between GC12 and GC3) to further investigate the extent of the influence of natural selection and mutational pressure on the codon usage bias of forage legumes. In Fig. 4, the slopes of the regression lines for the nine forage genomes were 0.2348, 0.2644, 0.4121, 0.5505, 0.4357, 0.3431, 0.2938, 0.4642, and 0.1390, and the correlation between GC12 and GC3 was weak (*r*_1_ = 0.159, *r*_2_ = 0.164, *r*_3_ = 0.267, *r*_4_ = 0.327, *r*_5_ = 0.248, *r*_6_ = 0.222, *r*_7_ = 0.189, *r*_8_ = 0.325, *r*_9_ = 0.088). This finding indicated that mutational pressure accounted for only 23.48%–55.05% of the codon usage patterns of the nine forage genomes, and factors such as natural selection accounted for 44.95–76.52%. Here, natural selection plays a very important or even dominant role in codon usage patterns in the nine forage legumes, which is consistent with the results of ENC-GC3s and PR2-plot analyses.

#### COA

**Fig. 5:**
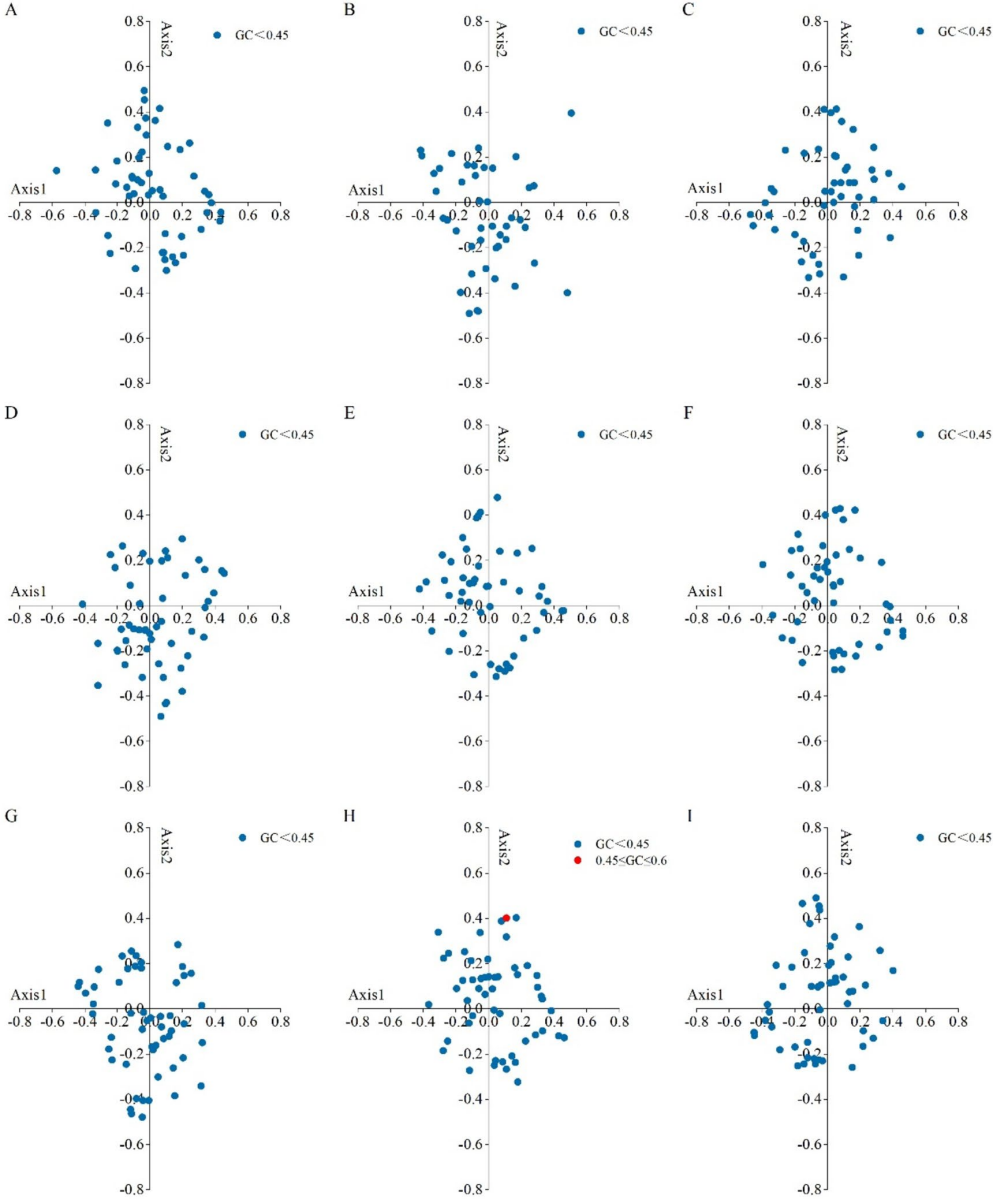
Correspondence analysis of the chloroplast genomes of nine forage legumes. (**A** *T. repens*, **B** *M. officinalis*, **C** *G. orientalis*, **D** *C. ternatea*, **E** *A. laxmannii*, **F** *G. officinalis*, **G** *P. sativum*, **H** *S. guianensis*, **I** *M. sativa*)

COA is a multivariate statistical method used for the relationship between variables in a sample. In this study, COA using RSCU revealed the main factors affecting the formation of codon usage patterns in the chloroplast genomes of forage legume plants. In the COA of nine forage legumes (Fig. 5), the coordinate origin indicates the average RSCU of all genes relative to axes 1 and 2. The first four axes accounted for 42.22%, 42.82%, 41.18%, 44.04%, 42.04%, 42.70%, 40.57%, 41.43%, and 42.44% of the overall variation. Axis 1 accounted for 19.20%, 16.45%, 19.46%, 19.77%, 18.67%, 20.11%, 18.12%, 19.07%, and 18.95% of the total variation in the nine forage legumes and thus was the major source of variation, accounting for approximately 20% of the total variation. This result suggested that codon usage may not be influenced by a single factor. Each gene in the chloroplasts of nine legume species was color-coded in different colors on a plane with axis 1 as the horizontal coordinate and axis 2 as the vertical coordinate to investigate the effect of GC content on CUB (Fig. 5). Among the nine legume species, only one gene in *S. guianensis* with a GC content of between 45 and 60% was color-coded in red, and all the other genes had a GC content of less than 45%. This high degree of consistency indicated that the sources of codon variation in the chloroplast genomes of the nine forage legumes are extremely similar.

**Fig. 6:**
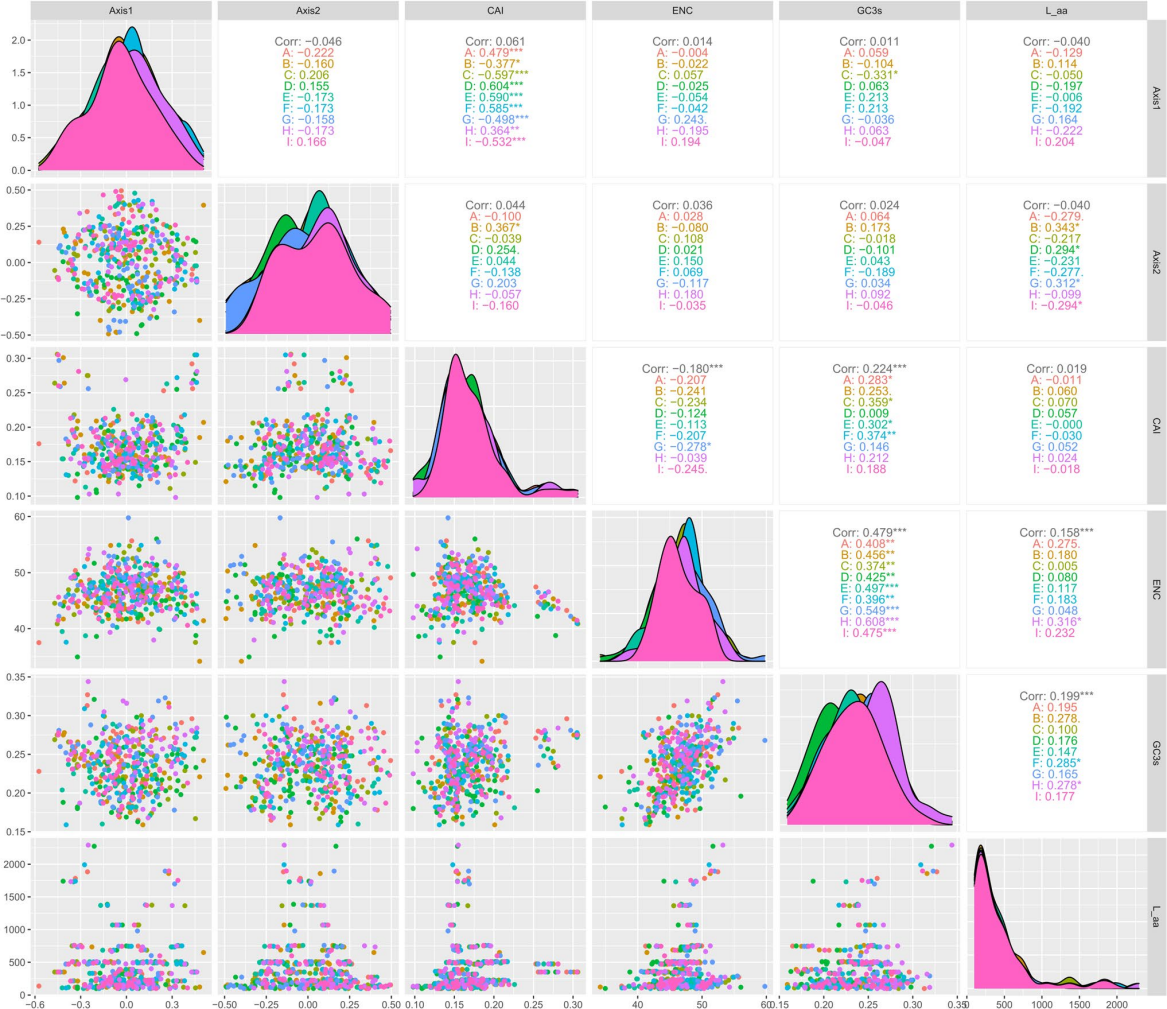
Correlation analysis of axis 1, axis 2 and codon usage index of the chloroplast genomes of nine forage legumes. (**A** *T. repens*, **B** *M. officinalis*, **C** *G. orientalis*, **D** *C. ternatea*, **E** *A. laxmannii*, **F** *G. officinalis*, **G** *P. sativum*, **H** *S. guianensis*, **I** *M. sativa*)

Correlation analysis was performed on axis 1, axis 2 and codon index to further explore the factors affecting codon use preference (Fig. 6). The results showed that the CAI of *G. orientalis*, *P. sativum*, and *M. sativa* had a highly significant negative correlation with axis 1, the CAI of *M. officinalis* showed a significant negative correlation with axis 1; the CAI of *T. repens*, *C. ternatea*, *A. laxmannii*, *G. officinalis* and *S. guianensis* showed a highly significant positive correlation with axis 1; the L_aa of *M. officinalis*, *C. ternatea*, and *P. sativum* showed a significant positive correlation with axis 2, the L_aa of *M. sativa* showed a significant negative correlation with axis 2, *T. repens*, *G. orientalis*, *A. laxmannii*, *G. officinalis* and *S. guianensis* showed a nonsignificant correlation with axis 2. These findings indicated that gene expression and gene length have an effect on the codon bias of the nine forage legumes, with a greater effect on gene expression.

### Phylogenetic relationships

**Fig. 7:**
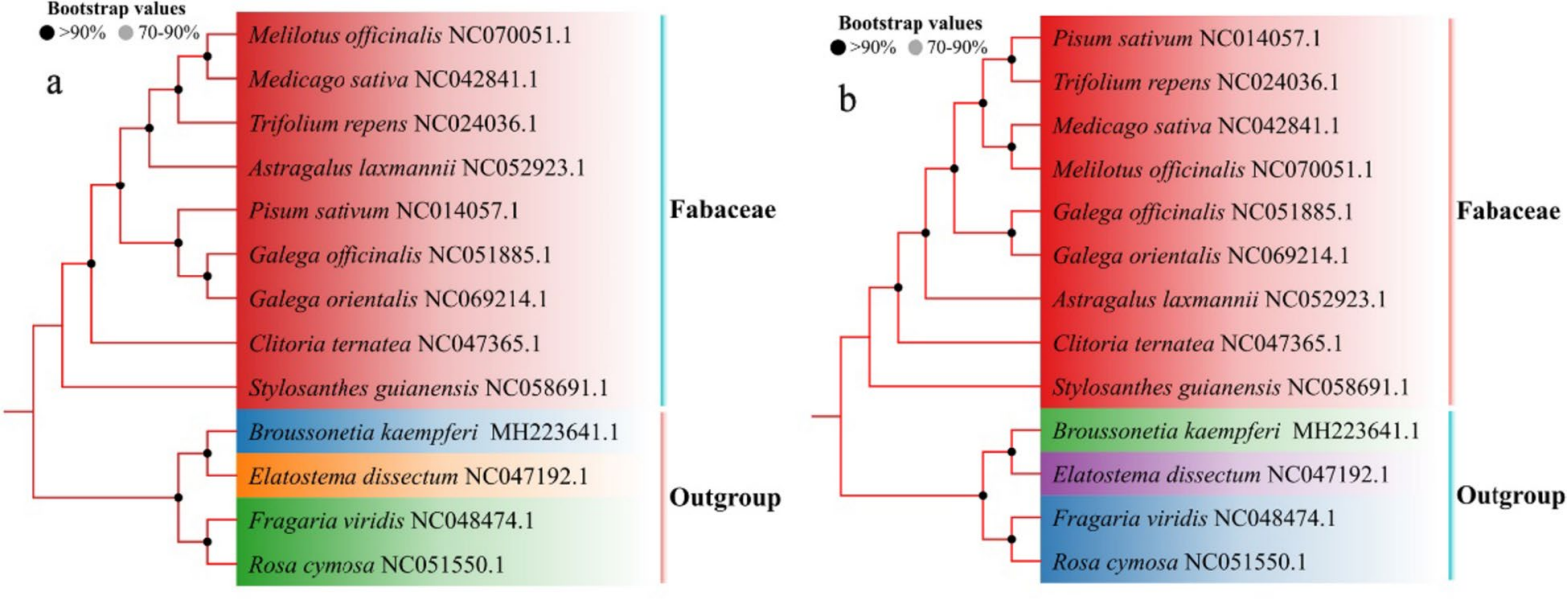
Phylogenetic tree based on the complete chloroplast genome (**a**) and protein coding sequences (**b**)

In phylogenetic trees, species are clustered together according to different genera or proximity of kinship, and the nodes of the constructed phylogenetic tree, all have very high support. The phylogenetic relationships based on the chloroplast genome (Fig. 7a) and protein-coding genes (Fig. 7b) were highly similar. The nine forage legumes constituted a monophyletic clade, which significantly differed from the outgroups. Most clades were strongly supported by high BS and posterior probabilities (PP). In both phylogenetic trees, the nine forage legumes were categorized into three major clades. The first clade consisted of *M. officinalis*, *M. sativa*, *T. repens*, *A. laxmannii*, *P. sativum*, *G. officinalis*, and *G.orientalis*. *C. ternatea* and *S.guianensis* formed the second and third clade, respectively. A sister relationship was observed between *M. officinalis* and *M. sativa*, and between *G. officinalis and G. orientalis*, and this relationship was strongly supported (BS = 100 and PP = 100). *S. guianensis* was distantly phylogenetically related to the remaining eight forage legumes.

## Discussion and conclusion

Codon usage bias is an important feature of genome evolution, and is significant for the study of plant genetic engineering and genetic evolution (Liu et al. 2020). The chloroplast genomes of dicotyledonous plants prefer codons ending in A or U, whereas monocotyledonous plants prefer codons ending in G or C (Murray et al. 1989). The frequency of synonymous codon use varies among organisms, and GC composition, tRNA abundance, gene expression, and gene length are the key factors influencing codon use bias (Niu et al. 2021). Several studies focused on the codon usage preference and its causes in various plants, such as *Camellia japonica* (Wang et al. 2022), *Gynostemma pentaphyllum* (Zhang et al. 2021), and *Manihot esculenta* (Geng et al. 2022).

The codon usage pattern is closely related to the GC content. In this study, we calculated the GC content of the three loci of the forage chloroplast genome codons GC1, GC2, and GC3 to explore the codon usage preference pattern. The nine forage legumes codons had the highest percentage of GC1 and the lowest percentage of GC3, and the average GC content was below 40%. This finding indicated that the chloroplast genes of the nine forage legumes preferred codons ending in A/T, which was consistent with previous studies. The chloroplast genomes of other plants, such as *Panicum incomtum* (Li et al. 2021), *Oryza australiensis* (Chakraborty et al. 2020), and *Euphorbia esula* (Wang et al. 2020) also tend to use codons ending in A or T. RSCU analysis revealed that most of the optimal codons (RSCU > 1) of the nine forage legumes ended in A/T, and the less common codons (RSCU < 1) ended in G/C. This finding is consistent with the results of the base composition analysis. Analysis of GC content indicated that among the nine forage legumes, *T. repens* was more similar to *M. officinalis*, *A. laxmannii*, and *G. officinalis* in terms codon preference. Hence, these species were presumed to be closely related. Comparative analysis of RSCU values revealed that the nine forage grasses had 29 common preferred codons, of which 96.68% ended in A/T.

Codon usage preference is mainly influenced by natural selection and mutational pressure (Rao et al. 2011). However, the main factors of codon usage preference differ among species. COA of the three codon sites in a neutral plot revealed that when mutational pressure plays a dominant role, the trends of base composition changes in the three codon sites should be similar. On the contrary, when natural selection plays a dominant role, there is no correlation between the three codon sites (Sharp et al. 2010). In our study, no significant correlation was found between GC12 and GC3 in the chloroplast genomes of the nine forage legumes. Mutation pressure accounted for 23.48%–55.05% of the codon usage pattern, and factors such as natural selection accounted for 44.95%–76.52%, suggesting that natural selection may have influenced codon selection in forage grasses. The slopes of the regression lines indicated that natural selection played a dominant role in the formation of codon usage patterns in constructing the codon bias of the forage chloroplast genomes. In conclusion, natural selection played a dominant role in determining codon usage bias in the chloroplast genomes of the nine forage legumes, followed by mutational pressure. This finding is similar to previous studies on the chloroplast genes of *P. sativum* (Bhattacharyya et al. 2019), *Medicago truncatula* (Song et al. 2018), and *Elaeis guineensis* (Aditama et al. 2020).

The PR2-plot showed that the frequency of use between A/T and G/C in codon position 3 of nine forage legumes was not uniform in the number of genes distributed in the four quadrants. Most genes were located below the midline in the vertical direction, and the number of genes on the right side of the midline was higher than that on the left side of the midline in the horizontal direction. Therefore, the frequency of G and T in the third position of the codon is higher than that of C and A. Natural selection is the main factor of codon usage bias in the chloroplast genomes of nine forage legumes (Zhang et al. 2007).

Neutral plot analysis, PR2-plot analysis, and ENC plot analysis revealed that the codon preferences of the chloroplast genomes of the nine forage legumes were jointly affected by natural selection and mutational pressure, with natural selection playing a dominant role. The result that natural selection is the main driver of chloroplast genome was also observed in other plants such as *Miscanthus* (Sheng et al. 2021), *P. sativum* (Bhattacharyya et al. 2019), and *M. truncatula* (Song et al. 2018). On the basis of these results, different genomes may be affected by different pressures. A total of 149 optimal codons were found in the nine forage legumes evaluated in this paper, with GCT, ACT, CCT, GGT, and TCT as the common ones. These results are important for improving the expression of chloroplast genes in host cells. The selection of heterologous expression hosts directly affects the expression of imported genes in host organisms. To realize the successful expression of exogenous genes and improve their expression, we should select the ones with few differences in codon usage preference as the receptor. By comparing the differences in the codon usage frequency of the nine forage genes and the four model organisms, we have found that *S. cerevisiae* is the best heterologous expression host.

The study of plant phylogeny from the perspective of chloroplast whole genome has become one of the hotspots in current plant classification research. In this study, the phylogenetic relationships based on the chloroplast genome and protein-coding genes were highly similar. The nine forage legumes constituted a monophyletic clade, which significantly differed from the outgroups, and most clades were strongly supported by high BS and PP.

### Supplementary Information

Below is the link to the electronic supplementary material.

Supplementary file1 (RAR 2895 kb)

Supplementary file2 (RAR 105 kb)

## Data Availability

Data will be made available on request.
